# What does global mean temperature tell us about local climate?

**DOI:** 10.1098/rsta.2014.0426

**Published:** 2015-11-13

**Authors:** Rowan Sutton, Emma Suckling, Ed Hawkins

**Affiliations:** National Centre for Atmospheric Science, Department of Meteorology, University of Reading, Reading, UK

**Keywords:** climate, variability, detection, attribution

## Abstract

The subject of climate feedbacks focuses attention on global mean surface air temperature (GMST) as the key metric of climate change. But what does knowledge of past and future GMST tell us about the climate of specific regions? In the context of the ongoing UNFCCC process, this is an important question for policy-makers as well as for scientists. The answer depends on many factors, including the mechanisms causing changes, the timescale of the changes, and the variables and regions of interest. This paper provides a review and analysis of the relationship between changes in GMST and changes in local climate, first in observational records and then in a range of climate model simulations, which are used to interpret the observations. The focus is on decadal timescales, which are of particular interest in relation to recent and near-future anthropogenic climate change. It is shown that GMST primarily provides information about forced responses, but that understanding and quantifying internal variability is essential to projecting climate and climate impacts on regional-to-local scales. The relationship between local forced responses and GMST is often linear but may be nonlinear, and can be greatly complicated by competition between different forcing factors. Climate projections are limited not only by uncertainties in the signal of climate change but also by uncertainties in the characteristics of real-world internal variability. Finally, it is shown that the relationship between GMST and local climate provides a simple approach to climate change detection, and a useful guide to attribution studies.

## 1. Introduction

The subject of climate feedbacks focuses attention on global mean surface air temperature (GMST) as the key metric of climate change. GMST is also the central metric around which UNFCCC discussions of mitigation policy are based. However, GMST is a remote concept to most people. Fundamentally, it is changes on regional and local scales that affect people directly. Knowledge about changes on these smaller scales is essential for the development of robust adaptation strategies, and is also required by national policy-makers when debating mitigation options. From these considerations follows a simple but important question: what does knowledge of past and future GMST tell us about local climate?

The issue of the relationship between changes in GMST and other aspects of a changing climate has recently gained prominence in discussions of the so-called ‘pause’ or ‘hiatus’ in global mean surface temperature rise [1–4]. Over the period 1998–2013 there was little change in GMST, but other important variables showed continuing changes. For example, global mean sea level continued to rise, ocean heat content and hot temperature extremes continued to increase, and Arctic sea ice extent showed substantial declines [5–8]. This example illustrates that, at least over a period of a decade or two, changes in important aspects of climate may not be closely related to changes in GMST.

**Figure 1.**
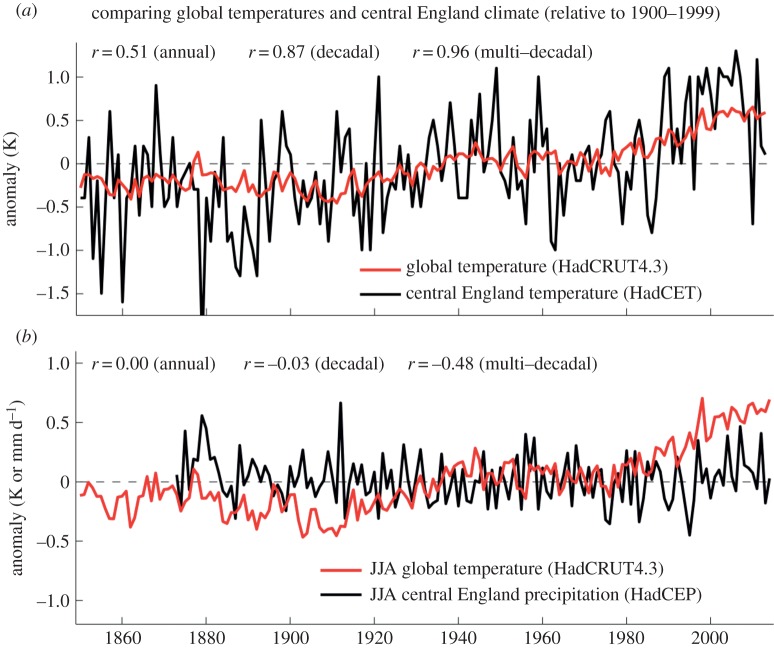
Comparison of global mean surface temperature anomalies (black, [9]) with annual mean central England Temperature ((*a*), red, [10]), and JJA mean central England precipitation ((*b*), red, [11]) anomalies. Correlations between the two time series in each case are shown for annual, running decadal and running multi-decadal means.

Another example is provided by figure 1, which shows a comparison between changes in GMST since 1850 and changes in annual mean temperature and summer precipitation in central England. On interannual timescales temperatures in central England show much greater variability and are not closely related to variations in GMST. By contrast, on multi-decadal timescales there is evidence of common behaviour, with a correlation coefficient of 0.96 between the two time series. Such a high correlation is quite surprising considering that central England represents just 0.005% of the Earth’s surface area. However, no such common behaviour is apparent in the record of summer precipitation. In this case the correlation on multi-decadal timescales is −0.48, so GMST explains less than 25% of the variance on these timescales. Clearly, the value of GMST as a predictor of local climate depends on both the variable and the timescale of interest.

The aim of this paper is to explore more systematically the relationship between changes in GMST and changes in local climate in different parts of the world. To do so requires consideration of both the internal variability in the climate system and the response of the climate system to changing forcing, such as that caused by the rapid increase in greenhouse gas concentrations in the atmosphere. Climate model simulations are used to separate the contributions from these two factors, and to explore their combination in reality. The structure of the paper is as follows. Section 2 examines the relationship between GMST and local climate in observational records. Sections 3 and 4 focus on model results and discuss internal variability and forced responses, respectively. Section 5 provides a synthesis and discussion, and conclusions are provided in §6. The focus in terms of timescales is decadal-to-multi-decadal. The motivation for this choice is that it corresponds to the timescale of anthropogenic climate change and instrumental records. In addition, figure 1 has already provided evidence of interesting relationships between GMST and local climate on these timescales.

## 2. Observations

We examine historical variations in surface air temperature (SAT) using data from the Berkeley Earth Surface Temperature (BEST) project [12]. BEST analyses weather station data to produce an estimate of land surface temperature changes from 1753 onwards. Their approach maximizes the use of short weather records and uses kriging techniques to produce temperature estimates with smaller uncertainties and wider spatial coverage than other datasets. The land data are combined with HadSST3 data [13] over the oceans to generate a globally complete dataset.

**Figure 2.**
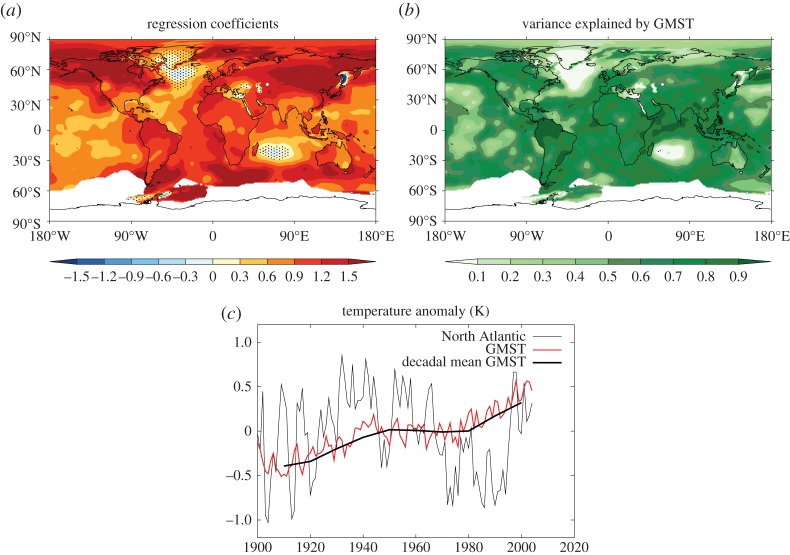
Linear regression of observed decadal mean local SAT on GMST. Regression coefficients (non-dimensional) (*a*), fraction of variance of local SAT explained by GMST (*b*) and comparison of time series for GMST and SAT in the North Atlantic Subpolar Gyre region (the ‘warming hole’) (*c*) are shown for the BEST observations over the period 1900–2013. Stippling in the regression pattern indicates regions where the 1-sigma uncertainty on the regression coefficient is as large, or larger than the coefficient itself.

Linear regression of local annual mean SAT on GMST over the instrumental record (figure 2) reveals that variations in GMST account for more than half the variance in local SAT on decadal and longer timescales over most of the planet, including much of the tropics.^1^ As we will discuss later, this is an interesting and far from obvious result (see §5).

The regression pattern generally shows higher values (greater than 1 K local SAT per degree of GMST) over land areas than sea areas. Higher values are also found in the Arctic and in the South Atlantic and southern Indian Oceans. Two regions where low values are found stand out: the subpolar North Atlantic and the Indian Ocean east of Madagascar. In both of these regions GMST accounts for less than 20% of the variance on decadal timescales. The North Atlantic region has been described as a ‘warming hole’ [14]. Figure 2 shows that, compared with GMST, surface temperatures in this region warmed much more rapidly in the early twentieth century, then cooled very rapidly in the 1970s before warming very rapidly in the 1990s. This behaviour is widely described as ‘Atlantic Multidecadal Variability’ or ‘AMV’. Potential drivers of AMV include the variations in the Atlantic Meridional Overturning Circulation (AMOC), which may be internal or forced, and external forcing factors such as greenhouse gases, anthropogenic aerosols and volcanic eruptions. However, there is currently no consensus on the relative importance of these various drivers [14–17].

Inspection of the SAT time series for the Indian Ocean region shows an abrupt warming in the late 1940s (not shown), which is not seen in GMST. However, this is a period when very little *in situ* data were available for this region, which suggests that this warming may not be real, and that the apparent differences from the evolution of GMST are similarly unreliable. These data issues merit further research.

Further interpretation of the observational results shown in figure 2 requires the use of model results to understand the characteristics of internal variability and forced responses; these are discussed in the next two sections, followed by a synthesis in §5.

## 3. Internal variability

Internal variability refers to fluctuations in climate that arise from instabilities in one component of the climate system (e.g. baroclinic instability in the atmosphere or ocean) or from interactions between different components (e.g. the El Niño Southern Oscillation, ENSO). These fluctuations arise even in the absence of any change in forcing. Some internal variability (of which ENSO is an example) causes significant fluctuations in GMST, but most does not. However, internal variability plays a central role in explaining climate variability on regional and smaller scales [18,19].

On interannual and shorter timescales, internal variability exhibits some preferred timescales (e.g. intra-seasonal variability associated with the Madden Julian Oscillation and interannual variability associated with ENSO). It is not known whether preferred timescales of internal variability also exist on decadal or longer timescales. There is undoubtedly memory in the oceans and land surface (e.g. arising from ground water, permafrost and vegetation) on decadal-to-centennial timescales, and memory in the cryosphere extends to much longer timescales, but it is unclear whether such memory leads to significant peaks in the power spectrum of internal variability or merely causes a broader enhancement of power at low frequencies (i.e. reddening the power spectrum).

**Figure 3.**
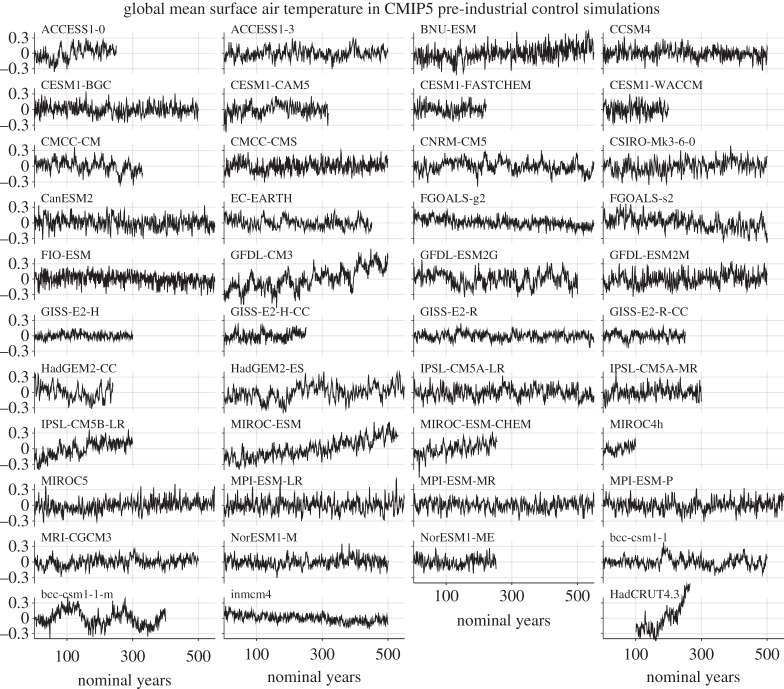
Internal variability in annual mean GMST anomalies (K) for CMIP5 pre-industrial control experiments. HadCRUT4 [9] observations are also shown for comparison (bottom right). The same vertical scale is used for all panels.

The study of internal variability on decadal and longer timescales is hampered by short observational records and difficulties in separating internal variability from forced signals in the face of substantial uncertainties in both forcings and responses. Climate model simulations offer an alternative approach to study internal variability in more controlled circumstances, but a difficulty here is that different models show surprisingly diverse behaviour [20,21]. Figure 3 illustrates internal variability in GMST in CMIP5 model control simulations (i.e. simulations under steady forcing). Models differ by a factor of 3 in standard deviation (9 in variance, *after* detrending) and also differ in the shape of their power spectra. Some models (e.g. GFDL-CM3) show evidence of preferred timescales while others do not. The magnitude of internal decadal variability in GMST is influenced by climate feedbacks, and is directly relevant to understanding events such as the recent ‘hiatus’ in GMST rise, discussed in §1. Thus deciding which models provide the most accurate representation of internal variability in the real world is a key challenge, and is closely linked to the challenge of constraining climate feedbacks. Note that differences in internal variability among models are even greater on regional scales [20,21]. Note also that none of the unforced models shown in figure 3 exhibits a centennial trend as large as that observed (in spite of the fact that some of the models exhibit significant drifts), which is evidence for the importance of forced signals in the real world (see §§4 and 5).

**Figure 4.**
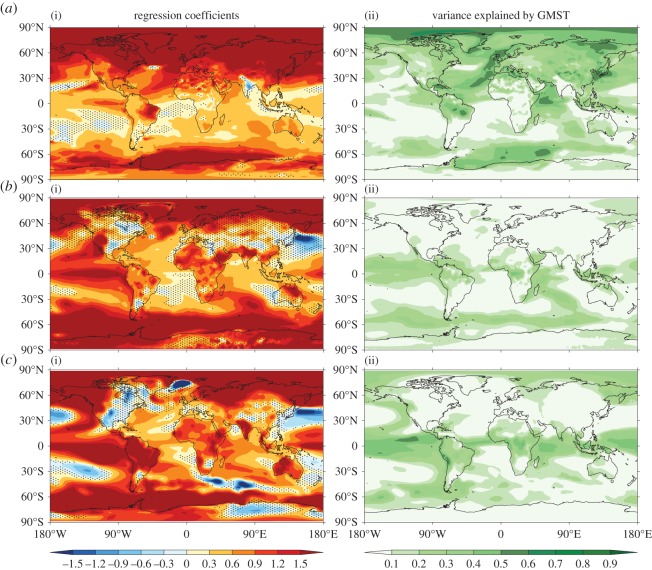
Linear regression of local decadal mean SAT on GMST. Regression coefficients (i) and fraction of variance of local SAT explained by GMST (ii) for CMIP5 pre-industrial control simulations are shown from HadGEM2-ES (*a*), CCSM4 (*b*) and CanESM2 (*c*). Stippling indicates regions where the 1-sigma uncertainty on the regression coefficient is as large, or larger, than the coefficient itself. (Further results for other models may be inspected at: http://www.met.reading.ac.uk/~ed/EMMA/index2.html.)

Regression analysis can be used to identify the spatial patterns of climate change that are related to internal variability in GMST [22]. Application to CMIP5 control simulations (figure 4) reveals that:

— In all models, over most of the planet, GMST accounts for less than 20% of the variance in local SAT on decadal timescales (and less than 10% of the variance in local decadal mean precipitation, not shown). This finding is consistent with our expectation that most internal variability is unrelated to GMST.
— The regions where GMST accounts for more than 50% of the variance in local SAT differ substantially between models. For example, in some models the relevant regions are found in the tropics, while in other models higher latitudes dominate [22]. This finding illustrates further the diversity of current climate models in their simulation of internal decadal variability. Note also that decadal variability may be influenced by lower frequency variability and therefore exhibit non-stationary characteristics, as is the case for interannual ENSO variability [23].

## 4. Forced responses

**Figure 5.**
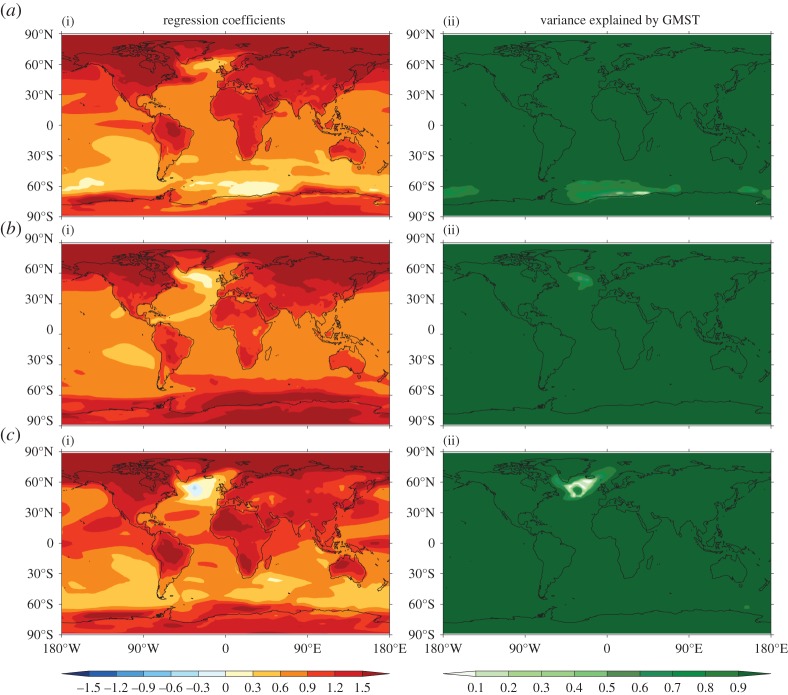
Linear regression of local decadal, ensemble mean SAT on GMST in CMIP5 RCP8.5 simulations over the period 2006–2100. Regression coefficients (i) and fraction of variance of local SAT explained by GMST (ii) are shown for HadGEM2-ES (*a*, 4-members), CCSM4 (*b*, 6-members) and CSIRO-Mk3.6.0 (*c*, 10-members). Stippling indicates regions where the 1-sigma uncertainty on the regression coefficient is as large, or larger than the coefficient itself.

To compare cleanly the forced responses simulated by different climate models it is necessary to remove, or minimize, the effects of internal variability. Where they are available ensemble simulations, in which individual ensemble members differ in their realization of internal variability, provide a very valuable resource [19,24]. In particular, the ensemble mean provides an improved estimate of the forced response. Figure 5 shows results from regression of ensemble mean local SAT on ensemble mean GMST for three different CMIP5 climate models, all under the RCP8.5 future forcing scenario [25]. Note that regression on GMST effectively removes the influence of inter-model differences in (transient) climate sensitivity (governed by feedbacks) on the magnitude of warming for given forcing, so that attention can be focused on the spatial patterns of response. The regression patterns for different models show a high degree of similarity and exhibit the major features that are well known from IPCC reports, such as enhanced warming over land and over the Arctic. In these analyses GMST accounts for over 90% of the variance over almost all the planet. The exceptions are associated either with regions of particularly large variability or with a nonlinear response to rising GMST. The North Atlantic subpolar gyre (NASPG) region, which stands out in several models, is an example of the latter. Changes in surface temperature in this region are influenced by a slowdown in the AMOC [14]. The magnitude and timing of this slowdown are very uncertain [26]. In some models the nonlinear relationship to GMST reflects an initial cooling of the NASPG, presumably linked to an AMOC slowdown, followed by a warming as the continued rise in greenhouse gas forcing comes to dominate (not shown). Another possibility is a gradual slowdown initially, followed by an abrupt shut-down [27]. While such nonlinear responses appear to be moderately rare, they present an obvious limitation on the use of ‘pattern scaling’ approaches for the generation of climate projections [28,29].

A comparison of figure 5 with figure 4 shows that the spatial pattern of greenhouse gas forced SAT response differs from that associated with internal variability in GMST (notwithstanding some common features, e.g. enhanced warming over the Arctic). A consequence is that the spatial pattern of internal variability cannot be used as a reliable way to estimate the forced response pattern.

**Figure 6.**
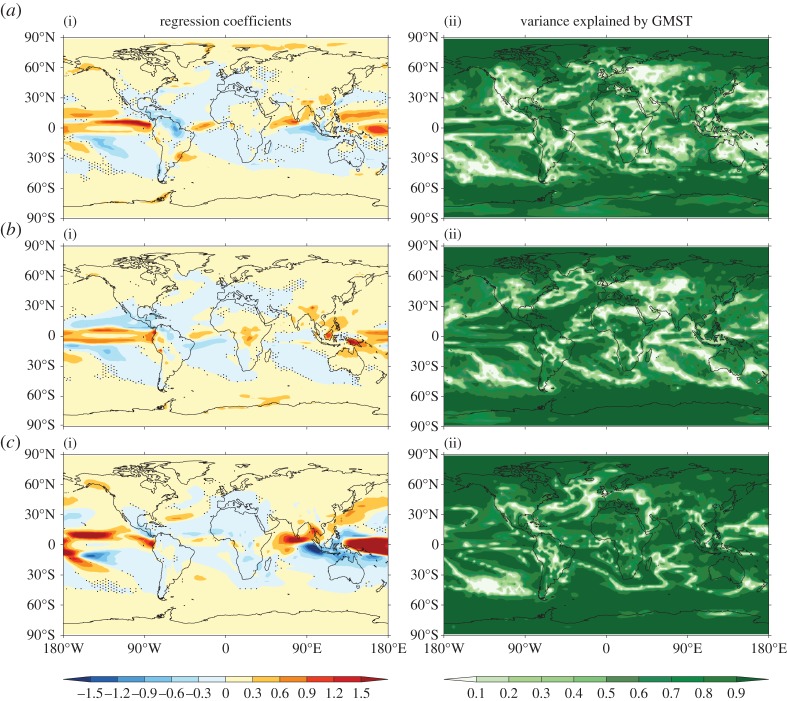
Linear regression of local decadal ensemble mean precipitation on GMST in CMIP5 RCP8.5 simulations over the period 2006–2100. Regression coefficients (i) and fraction of variance of local precipitation explained by GMST (ii) are shown for HadGEM2-ES (*a*, 4-members), CCSM4 (*b*, 6-members) and CSIRO-Mk3.6.0 (*c*, 10-members). Stippling indicates regions where the 1-sigma uncertainty on the regression coefficient is as large, or larger than the coefficient itself.

The patterns of forced response in precipitation change show much greater diversity between models than is the case for SAT change (figure 6). This ‘response uncertainty’ [30], which is notably high in the tropics, is primarily related to dynamical rather than thermodynamic aspects of the response [31]. Changes in heavy precipitation—which are more strongly controlled by thermodynamics—appear to be more robust than changes in mean precipitation [32]. Considering individual models, GMST still accounts for over 90% of the variance in local mean precipitation over large regions of the planet. Most of the exceptions are regions where the forced response is small, so internal variability still influences the ensemble mean (given the modest size of the ensembles—see figure 6, caption). However, there are also regions where changes in local precipitation are not simply proportional to changes in GMST. For example, several CMIP5 models project non-monotonic changes in precipitation over tropical South America in boreal summer, associated with a projected southward migration of the ITCZ [33].

Figures 5 and 6 focus on the response to greenhouse gas forcing. A further issue for understanding the relationship between GMST and local climate is that of competition between radiative forcings. For example, during the twentieth century some of the warming effect of increasing greenhouse gas concentrations was offset by the cooling effect of increasing anthropogenic aerosols [34]. In principle the responses to two forcings of opposite sign can cancel out entirely leading to no net change in GMST, a recognition which has stimulated recent interest in Solar Radiation Management (SRM) approaches to geo-engineering [35]. However, a basic but fundamental point is that no net change in GMST does *not* imply no change in climate. On the contrary, different forcings may lead to substantially different patterns of regional climate change [34,36]. So, for example, forcings that result in no net change in GMST may, nevertheless, cause substantial changes in the regional-scale hydrological cycle [37]. The contribution of anthropogenic aerosol forcing to the onset of severe drought in the African Sahel in the 1970s may be an example of such competition [38]. Note that competition between greenhouse gas and aerosol forcing, together with large uncertainties in the magnitude of the aerosol forcing, is a key reason why the interpretation of model simulations of the instrumental period (often termed ‘historical simulations’) is more complicated than might be expected.

## 5. Combining internal variability and forced responses

In the real world, the forced response of anthropogenic climate change is gradually emerging from the background of natural internal variability. At the local scale, internal variability is the dominant factor in people’s experiences of day-to-day and year-to-year variations in the weather. Individuals and societies are adapted to a certain level, or range, of weather variability that is characteristic of the places where they live, and the same is true of natural ecosystems. It is when climate change leads to weather or climate events that are outside of this familiar range of experience that large and costly impacts typically arise [39,40]. These considerations highlight the importance of assessing not only the absolute magnitude of climate change but also its magnitude relative to natural internal variability, at a local scale. From this perspective, the rate of climatic warming is fastest in the tropics and summer season even though the absolute magnitude of warming is greater at higher latitudes [20,30,41]. Interestingly, uncertainties in projecting the emergence of the climate change signal are determined almost as much by uncertainties in internal variability as by uncertainties in the signal itself [20].

The relative importance of the forced climate response and internal climate variability, and hence the emergence of the signal of climate change, depends on several factors: the variable of interest, the spatial scale, the temporal scale, and the time horizon [18]. We have seen that GMST is a powerful predictor of the forced climate response but provides little information about internal variability at the local scale. It follows that the value of GMST as a predictor of local climate also depends on these same factors. From the perspective of adaptation, the spatial scales of interest are regional-to-local and the variables and temporal scales of interest are typically set by considering a particular class of high impact event (heavy rainfall, hot days, drought, etc.). The remaining question is therefore: over what time horizon does the signal of climate change emerge from the background of climate variability? This question may also be stated as: over what time horizon is GMST a useful predictor of local climate?

**Figure 7.**
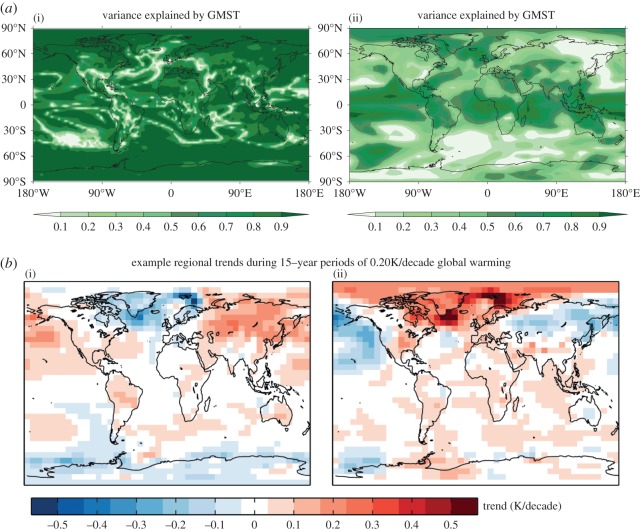
Analysis of simulations with the FAMOUS climate model under a scenario of 1% per annum increasing CO_2_ concentration. (*a*) Fraction of variance of local SAT explained by GMST from linear regressions of annual mean SAT on annual mean GMST over a time horizon of 140 years (i) and 20 years (ii) from a single ensemble member. (*b*) The diversity of SAT 15-year trend patterns from two simulations which both exhibit a trend in GMST of 0.20 K per decade.

Figure 7 shows an analysis of simulations with the FAMOUS climate model [42] under a scenario of 1% per annum increasing CO_2_ concentration. For a single simulation, over a time horizon of 140 years GMST accounts for 80% or more of the variance in local SAT over most of the planet. By contrast, over a time horizon of 20 years GMST accounts for less than 40% of the variance. The exact proportion will depend on the model examined, but an important robust result is that, on timescales of a few decades, at the local scale internal variability can easily dominate the forced response [18,19]. This finding is further illustrated in the lower panels of figure 7, which show maps of trends in SAT from two simulations which exhibit an identical trend in GMST (of 0.20 K per decade). The spatial pattern of SAT trend in the northern hemisphere is nearly opposite in these two realizations. For precipitation, the relative importance of internal variability is even greater (i.e. the signal to noise for precipitation change is lower [43]), with the result that local trends of many decades’ duration may be dominated by internal variability rather than the forced response [19,30,43].

The value of GMST as a predictor of local climate has relevance to detection and attribution of climate change [34]. As was noted in §2, the fact that GMST accounts for a large fraction (greater than 60%) of the observed variance in local SAT on decadal and longer timescales over most of the planet, is not an obvious result. Comparing figure 2 with figure 4 shows that GMST accounts for a much larger fraction of the observed variance in local SAT than would be expected if changes were governed entirely by internal variability. This result implies that decadal changes in SAT over the instrumental record have been substantially shaped by a forced response (figure 5). Thus figure 2 provides a simple form of climate change detection. This result could be formalized by developing a quantitative description of the characteristics of internal variability, either on a purely local basis or taking into account spatial patterns (e.g. figure 4). In view of the large diversity in simulated internal variability discussed in §3, such a description could not be very tightly bounded, but would nevertheless provide a null hypothesis against which the observed climate change signal could be tested.

Detection analysis does not identify which specific changes in forcing may have caused the observed climate change (the attribution problem), but ruling out internal variability as the sole or dominant cause is an important step. In addition, comparing local changes in climate on decadal timescales with changes in GMST provides a very simple approach to identify regions where particularly careful attention in attribution research may be required, as is undoubtedly the case for the NASPG region, discussed in §§2 and 4.

## 6. Conclusion

The value of GMST as a predictor of regional changes in climate depends on the relative importance of forced responses and internal variability, which depends in turn on the variable of interest; the spatial scale; the time scale; and the time horizon. Internal variability is increasingly important at smaller space and time scales and for shorter time horizons. It is also more important for mean precipitation than for SAT. In the absence of any forced climate signals (i.e. pure internal variability), GMST explains only a small fraction of the variance in local climate (typically less than 20% for decadal mean SAT). In the case of a pure forced signal, if the response is linear then GMST necessarily explains 100% of the variance in local climate. However, nonlinear responses to greenhouse gas forcing are known to exist, and are expected to be important in specific regions such as the North Atlantic Subpolar Gyre. Furthermore, competition between multiple forcings (e.g. greenhouse gases and anthropogenic aerosol) complicate the relationship between changes in GMST and changes in local climate. An important consequence is that large forced changes in regional climates may occur in the absence of any (*net*) forced change in GMST, a fact which is crucial to the assessment of geo-engineering proposals.

In reality, the signal of forced climate change on local scales is progressively emerging from the background of natural variability. Measuring rates of change relative to this background variability (rather than solely in absolute terms) is important for the assessment impacts and changing risks. Projections of emergence are limited by significant uncertainties in both the climate change signal on local scales (response uncertainty) and uncertainties in the characteristics of internal variability. Large differences in the latter characteristics have been found in comparisons between different climate models. For some applications, uncertainties in internal variability may be as important as the uncertainties in climate feedbacks.

In an analysis of observational records, it has been shown that GMST explains 60%, or more, of the variance in local SAT on decadal and longer timescales over a large fraction of the planet. When considered in the context of other findings, this result implies that decadal changes in SAT over the instrumental record have been substantially shaped by a forced response. Thus analysing the relationship between GMST and local climate provides a simple approach to climate change detection, and a useful guide to attribution studies.

## Footnotes

1 GMST accounts for more than 60% of the variance in local SAT over 58% of Earth’s surface area. A similar analysis of (non-interpolated) HadCRUT4 data [9] shows that GMST accounts for a similarly high fraction of the variance in regions for which there is data coverage over most of the twentieth century (not shown).

## Data accessibility

Data analysed in this paper is publically available at the following repositories. Observational datasets: HadCRUT4 temperature data (Climatic Research Unit, University of East Anglia) http://www.cru.uea.ac.uk/cru/data/temperature/; Central England Temperature records (UK Met Office) http://www.metoffice.gov.uk/hadobs/hadcet/data/download.html; UK regional precipitation records (UK Met Office) http://www.metoffice.gov.uk/hadobs/hadukp/data/download.html; Berkeley Earth temperature (land and ocean) data (BEST) http://berkeleyearth.org/data/. Model simulations: CMIP5 piControl and RCP8.5 simulations (PCMDI database) http://cmip-pcmdi.llnl.gov/cmip5/data_portal.html. Figures 3–7, along with those from other models from the CMIP5 and FAMOUS experiments can be further explored at: http://www.met.reading.ac.uk/~ed/EMMA/index2.html.
